# Magneto-electric Nanoparticles to Enable Field-controlled High-Specificity Drug Delivery to Eradicate Ovarian Cancer Cells

**DOI:** 10.1038/srep02953

**Published:** 2013-10-16

**Authors:** Rakesh Guduru, Ping Liang, Carolyn Runowicz, Madhavan Nair, Venkata Atluri, Sakhrat Khizroev

**Affiliations:** 1 Center for Personalized NanoMedicine, Department of Immunology, Herbert Wertheim College of Medicine, Florida International University, Miami, Florida 33199; 2 Electrical and Computer Engineering, College of Engineering, Florida International University, Miami, Florida 33174; 3 Electrical Engineering, University of California, Riverside, CA 92521; 4 Department of Obstetrics and Gynecology, Herbert Wertheim College of Medicine, Florida International University, Miami, Florida 33199

## Abstract

The nanotechnology capable of high-specificity targeted delivery of anti-neoplastic drugs would be a significant breakthrough in Cancer in general and Ovarian Cancer in particular. We addressed this challenge through a new physical concept that exploited (i) the difference in the membrane electric properties between the tumor and healthy cells and (ii) the capability of magneto-electric nanoparticles (MENs) to serve as nanosized converters of remote magnetic field energy into the MENs' intrinsic electric field energy. This capability allows to remotely control the membrane electric fields and consequently trigger high-specificity drug uptake through creation of localized nano-electroporation sites. In in-vitro studies on human ovarian carcinoma cell (SKOV-3) and healthy cell (HOMEC) lines, we applied a 30-Oe d.c. field to trigger high-specificity uptake of paclitaxel loaded on 30-nm CoFe_2_O_4_@BaTiO_3_ MENs. The drug penetrated through the membrane and completely eradicated the tumor within 24 hours without affecting the normal cells.

The development of a technology that is capable of high-specificity targeted delivery of anti-neoplastic drugs would be a significant breakthrough in cancer in general and ovarian cancer in particular. Although the circulatory system can deliver a drug to every cell in the body, delivering the drug specifically inside the tumor cell past its membrane without affecting the healthy cells remains a challenge^1^,^2^,^3^. In ovarian cancer, intraperitoneal (IP) delivery through a surgically implanted catheter has shown improved survival rates. However, catheter complications and toxicity have precluded widespread adoption of this invasive means of delivery^4^. Current research attempts to go around these limiting factors by using nanoscale systems^5^,^6^,^7^. Often, as immunological reagents, monoclonal antibodies are used to recognize the tumor-specific biomarker while the nanoscale control further improves the specificity and targeted drug delivery capability in general^8^,^9^,^10^. Nonetheless, in spite of the tremendous progress in this field during the last decades, the capability of targeted delivery with adequately high specificity (to tumor cells) remains an important roadblock to finding a cure for cancer.

In this paper, we present a study in which we address this challenge through a new physical concept. It exploits (i) the difference in the electric properties of the membrane between the tumor and healthy cells and (ii) the ability of the recently discovered body-temperature magneto-electric nanoparticles (MENs) to function as nano-converters of remotely supplied magnetic field energy into the MENs' intrinsic electric field energy^11^,^12^,^13^. Like the conventional magnetic nanoparticles (MNs), MENs have a non-zero magnetic moment and therefore can be controlled remotely via application of an external magnetic field. However, unlike MNs, MENs offer a new far-reaching function, which is an energy-efficient control of the intrinsic electric fields within the nanoparticles by an external magnetic field. This unprecedented capability is a result of the strong magneto-electric (ME) coupling in this new class of nanostructures even at body temperature^11^,^12^,^13^. As a result, MENs introduced in a biological microenvironment act as localized magnetic-to-electric-field nano-converters that allow remote control and generation of the electric signals that underlie the intrinsic molecular interactions. Recently, we exploited this capability: (i) to achieve remotely-controlled brain stimulation in patients with Parkinson's Disease by applying low-energy a.c. magnetic fields to control the a.c. electric signals in the central nervous system (CNS) using intravenously injected MENs and (ii) to deliver and release on-demand (via an external field) anti-retroviral (ARV) drug AZTTP for treatment of HIV-1 reservoirs across the blood brain-barrier (BBB)^14^,^15^. In this study, we exploit this capability to achieve the field-controlled specificity of the drug-loaded MENs as required to significantly improve the state of chemotherapy.

The MEN's new capability to control the local electric fields remotely (via magnetic fields) opens an exciting and previously unexplored path to exploit the intrinsic electric properties of the cell membrane. Due to the presence of ion channels and other electric-field driven properties, the cell membrane is an electrically polarizable medium. As a result, its properties can be significantly affected by an electric field. In fact, electroporation is one such well-known characteristic that exploits the dependence of the membrane's porosity on the electric field^16^,^17^,^18^,^19^,^20^,^21^. The electroporation has been widely studied as a means to trigger drug delivery into the cells. Through macroscale studies (on samples with centimeter sizes) it is known that an electric field of higher than 1000 V/cm creates sufficiently large pores for the drug nanoformulations to penetrate through the membrane. Our new approach was to use MENs to exploit the promising delivery technique by scaling it down into the nanoscale. Due to this NANO-ELECTROPORATION, magnetic-field-activated MENs loaded with the drug and optionally with the biomarker-specific antibodies (for delivery to the tumor cells) can generate localized fields large enough to open up the membrane pores in their proximity only and thus let the drug inside the tumor cells. Because this process is relatively energy efficient, most of the energy goes to fulfill the main operation (of opening up the local pores, i.e. the nanoscale electroporation) and consequently it doesn't result in any significant and potentially damaging energy dissipation, e.g., in terms of heat. The interaction between the MENs and the electric system of the membrane effectively serves as a field-controlled gate to let the drug-loaded nanoparticles enter specifically the tumor cells only. An artist's view of the main hypothesis is presented in Fig. 1. In this case, the origin of the specificity to the tumor cells is two-fold. First, the biomarker-specific antibodies steer the drug-loaded MENs (to which they also are attached) to the tumor cell membrane. Second, even higher specificity is achieved due to the fact that the tumor and healthy cells have different values of the threshold field, H_th_, for the “gate” to open up. Indeed, it is well-known that the electric properties differ significantly between the healthy and tumor cells of the same type^22^. In general, the tumor cells have substantially lower values of the potential compared to that of the healthy cells. Consequently, the cancer cells must also have a significantly lower value of the threshold field for the drug-loaded MENs to enter the cell. Considering the value for the ME coefficient *α* ~ 100 mV cm^−1^ Oe^−1^, according to the simple isotropic expression for the ME effect, Δ*P* = *αH*, where *P and H* stand for the induced electric dipole field and the external magnetic field, respectively, the electric field of the order of 1000 V/cm can be generated a few nanometers away from the MEN merely by applying a magnetic field of 10 kOe. Moreover, the same order of magnitude electric can be generated by much smaller magnetic fields, of the order of 100 Oe, if one takes into account the edge effects because of the cubic symmetry of the real-life nanoparticles, as shown below. Due to the edge effect (significantly enhanced charge density at the edges), the electric field in the vicinity of MENs could be amplified by a factor of 100 or more depending on the proximity to the edge. Moreover, the edge effects in the case of ME materials might be even further enhanced because both the electric charge density and the effective magnetic charge density are amplified at the edges. Ideally, after the drug-loaded MENs penetrate into the cell cytosol through the “open” pores in the membrane, the drug can be released off the MENs by further increasing the field above the second critical value, H_r_, necessary for overcoming the drug-MEN binding energy. We described one underlying hypothesis of this field-controlled drug release process in a recent article^14^. This field strongly depends on the binding force between the MEN and the drug and consequently can be tuned in a large range through using different intermediate coating materials, field excitation frequencies and treatment durations. In summary, according to our idealistic hypothesis, there are two critical field values, H_th_ and H_r_, that define the drug penetration threshold through the tumor cell membrane and the following release of the drug into the cell cytosol, respectively. To ensure adequately high efficacy of the uptake, we need H_r_ > H_th_. To ensure the required specificity of the uptake to the cancer cells only, the external applied field, H_A_, needs to be higher than the release field for the tumor cells, H_r_cancer_, and lower than the threshold field for the healthy cells, H_th_healthy_. In summary, using MENs not only can provide field-controlled delivery but also can significantly improve the specificity to tumor (compared to the specificity defined by the monoclonal antibodies alone). When combined, monoclonal antibodies and MENs can make even a better delivery system. While the monoclonal antibodies steer the loaded drugs towards the surface of the tumor cells, the field-controlled MENs move the drugs across the cell membrane into the cytosol.

This new high-specificity nanotechnology can be applied to the treatment of cancer in general. In the current study, to prove our hypothesis, we used Epithelial Ovarian Cancer (EOC). EOC has been widely studied in the medical community^23^. Cytoreductive surgery followed by chemotherapy with mitotic inhibitor Paclitaxel (PTX) with platinum is the gold standard in treating EOC. In most cases, the drug administration is intravenous (IV). Less commonly the administration is IP. As noted, there are technical considerations and limitations to IP therapy, although it is more effective than IV therapy. In either case, the specificity of the drug uptake is still relatively low and as a result EOC remains a highly lethal malignancy. Therefore, the current study is relevant to this field. In addition, because of the high-specificity capability, the new nanotechnology can be used for targeted treatment of both localized and metastasized tumor cells. Finally, by its fundamental nature, this nanotechnology can be applied to a wide range of other cancers.

## Results

Through the described in-vitro studies on human ovarian carcinoma cell (SKOV-3) and healthy ovarian cell (HOMEC) lines, we demonstrated that high-specificity uptake of PTX-loaded 30-nm CoFe_2_O_4_@BaTiO_3_ MENs could be triggered by a low-energy 30-Oe d.c. remote magnetic field with relatively small or negligible heat dissipation^24^,^25^,^26^. Through kinetics studies we confirmed that the drug penetrated through the tumor cell membrane and eradicated the majority of the cells within a 24-hour period without affecting the surrounding healthy cells. Finally, to demonstrate the applicability of this nanotechnology to other cancers, we conducted a parallel study using a multidrug resistant (MDR) uterine sarcoma cell type MES-SA/DX5^27^,^28^,^29^.

The procedures to fabricate the nanoparticles with different sets of coatings and drug loadings are described in Section *Methods*. The release threshold field, H_r_, could be controlled in a wide range, from 10 Oe to substantially over 200 Oe, through different intermediate layers/coatings. A comparative analysis of the effect of the intermediate layer type on H_r _is summarized in Fig. S1. By default, in order to provide adequate coupling between the MENs and Flutax-2 (to provide the initial release field of the order of 30 Oe), before being loaded with the drug, the MENs were coated with 3-Angstrom thick glycerol monooleate (GMO) layers. The zeta-potential and size of the MENs, GMO-MENs, HER2-GMO-MENs, and PTX-GMO-MENs are shown is Table S3. For the purpose of a comparative analysis, we studied the following combinations of nanoparticles: (i) MENs loaded with PTX, (2) MENs loaded with PTX and the popular cancer biomarker HER-2 antibody, (3) free PTX, and (4) conventional MNs loaded with PTX. As the conventional MNs, 30-nm magnetite nanoparticles were used.

### Field-controlled drug release by MEN-based carriers

Drug release from these different MEN-based combinations was triggered by a magnetic field at different strengths and frequencies, according to the physics described in our earlier paper on the release of ARV drug AZTTP for treatment of HIV-1 virus in the brain^14^. The pellet obtained after the drug loading procedure was washed thrice with the phosphate-buffered saline (PBS) buffer, to remove any residual unbounded drug. The drug-loaded-MENs' pellet was added to 1 ml of the PBS buffer in a vial and subjected to a d.c. or a.c. magnetic field using a pair of Helmholtz coils connected to a d.c. or a.c. power supply, respectively. After exposing the vial to any magnetic field environment under study, the supernatant was obtained by spinning the sample at 3,000 rpm for 5 minutes and at 10°C. The supernatant was measured for the amount of the released drug spectrophotometerically through the absorbance at the PTX maximum wavelength of 230 nm^30^.

The results of the field-controlled drug release spectrophotometry (absorption) experiments are summarized in Fig. 2. Fig. 2A shows the percentage of the drug release after a 1-minute exposure to a magnetic field at three strengths, 12, 44, and 66 Oe, respectively, for three different frequencies, 0, 100, and 1000 Hz, respectively. As expected (see explanation above), for each frequency, there was a critical field, H_r_, at which the drug release was significantly boosted. The increase of the frequency in the range up to 1000 Hz under study increased the release efficacy (by over 40%) especially at the low field range. Fig. 2B illustrates the kinetics of the field-strength-frequency dependence of the release for the five values of the field exposure times, 1, 5, 10, 60, and 120 minutes, respectively. The quantitative values are also presented in Table S1. For every exposure time setting, a fresh solution with PTX-loaded GMO-coated MENs was used. The field-triggered drug release was also confirmed through atomic force microscopy (AFM), Fourier Transform Infra-Red (FTIR), mass spectrometry, and X-ray diffraction (XRD) pattern studies. The results are summarized in Fig. S2, Fig. S3, Fig. S4, and Fig. S5, respectively.

### Field-controlled drug uptake by tumor cells through the MEN-induced NANO-ELECTROPORATION

Fluorescent cellular drug uptake experiments were performed on the SKOV-3 cells using the four different drug forms under study, (i) free Flutax-2, (ii) Flutax-2 bound to the conventional MNs, (iii) Flutax-2 bound to GMO-MENs, and (iv) Flutax-2 bound to HER-2-GMO-MENs, respectively. The obtained Flutax-2 concentration was normalized to the protein amount. The results of the experiment performed in triplicates are shown in Fig. 3. These results showed that the drug uptake increased by a factor of five for the drug carried by field-controlled MENs compared to the drug driven by the HER-2 antibodies. The details of the related fabrication and measurements are described in Section *Methods*.

### Confocal microscopy to visualize the internal drug localization in SKOV-3 cell lines

To visualize the internal localization of each of the four drug forms under study, (i) free Flutax-2, (ii) Flutax-2-GMO-MENs, (iii) Flutax-2-HER-2, and (iv) Flutax-2-MNs, respectively, in SKOV-3 cell lines, we conducted the following fluorescence imaging experiments.

### Magnetic field dependence of drug uptake in cancer and healthy cells

To understand the field dependence of the described process, we performed the cellular drug uptake experiments under a varying magnetic field strength on both cancer ovarian (SKOV-3) and healthy ovarian cell (HOMEC) lines. The HOMEC cells were cultured according to the same procedures that are described for the SKOV-3 cells in Section *Methods*. As a control, the cells with GMO-MENs only (without Flutax-2) were treated under the equivalent conditions. The cell culture plates with the MENs and drug-GMO-MENs were exposed to three different field strengths, 5, 15, and 30 Oe, respectively. The results are summarized in Fig. 5. The measurements showed that as the field was increased above approximately 30 Oe, the drug penetration into the cancer cells (SKOV-3) greatly increased. On the other hand, it can be noted that the drug barely affected the healthy cells (HOMEC) in the field range under study.

### Cancer cell viability test

After we confirmed that the drug-loaded MENs in the vicinity of the cancer cells indeed acted as a field-controlled valve to let the drug in (due to the effective nano-electroporation effect according to our hypothesis), we studied the viability of the cancer cells for different combinations of the drug and the carrier after the drug penetrated through the cell membrane. (Here, maintaining the remote field at 30 Oe provided the specificity to the cancer cells or, in other words, ensured that the healthy cells were intact.) The confocal images that were obtained after a 24-hour field treatment are in Fig. 6. The three key combinations of the carrier included (i) no particle, (ii) HER-2-GMO-MENs (Note: Here, HER-2 stands for the HER-2 biomarker antibody), and (iii) GMO-MENs, respectively. Accordingly, the three images (from left to right) in Fig. 6A show the morphology of the cancer cells after 24-hour treatment by (i) the free drug (with no particle carrier), (ii) drug-HER-2-GMO-MENs with no field applied, and (iii) drug-GMO-MENs in a 30-Oe d.c. field. The three control images in Fig. 6B show the morphology of the cancer cells after the 24-hour treatment by the same three combinations of the carrier with no drug present. In addition, we conducted the confocal imaging and the trypan-blue cell viability tests on both SKOV-3 and HOMEC cell lines after 24- and 36-hour field treatment. The confocal images are shown in Fig. S6, while the tryphan-blue viability data are summarized in Table S2.

### In-vitro cytotoxicity assay

To determine the cytotoxicity of the GMO-MENs on SKOV-3 cells, a quantitative colorimetric XTT (sodium 2,3,-bis(2-methoxy-4-nitro-5-sulfophenyl)-5-[(phenylamino)-carbonyl]-2H-tetrazolium inner salt) assay was performed. The assay is based on the reduction of XTT tetrazolium salt by the viable cells to form orange colored formazan derivative. In this assay, 1 × 10^5^ cells were seeded per well in a 96-well plate and incubated at 37°C for 24 hours. After the incubation, the cell medium was replaced by the medium containing the GMO-MENs at a differential concentration of 0–100 μg/ml per well and the cells were incubated for another 24-hour period. Then, the cell medium was replaced with a fresh one and washed with the PBS buffer and cell viability assay was performed by adding 50 μl per well of XTT-activated solution from the XTT test kit supplied by ATCC and incubate for 4 hours at 37°C. The experiments were performed in triplicates. As summarized in Fig. S7, no significant cytotoxicity was observed.

### Heat-dissipation due to field-treatment with MENs

In this experiment, the temperature was measured locally via Infra-red (IR) camera FLIR-i3 on the surface of both cancer (SKOV-3) and healthy (HOMEC) ovarian cells before and after a field treatment, as shown in Fig. S8. The experimental error of the setup was approximately +/− 2 Celsius degrees of the infrared camera. The magnetic field of 30 Oe was applied for a 24-hour period. No significant heat dissipation was observed as a result of the field treatment. The negligible heat dissipation (compared to the conventional method) is a consequence of the intrinsic nature of the magneto-electric coupling which resulted in the relatively high high-efficacy control of intrinsic electric fields by external magnetic fields.

### Universal applicability: MENs-triggered drug uptake in MDR cell MES-SA/DX5

To demonstrate the applicability of the new nanotechnology to other cancers, we conducted a parallel study on a well-known multi-drug resistant cell line MES-SA/DX5. The results of the confocal microscopy imaging of the uptake of the same drug (Flutax-2) by this cell type is shown in Fig. S9. For comparison, the following four different drug-delivery-system combinations were studied: (a) no drug, (b) free Flutax-2, (c) Flutax-2-GMO-MENs with no field. (d) Flutax-2-GMO-MENs with 30 Oe field.

## Discussion

The results of the above experiments confirmed our hypothesis that MENs loaded with the drug PTX could serve as high-specificity remotely controlled (via magnetic fields) delivery nanosystems to treat EOC. We believe that this function was achieved due to the localized electroporation effects induced by the MENs in the vicinity of the cancer cell membranes when exposed to an external magnetic field. To refer to the effect at the nanoscale, we used the new terminology, “NANO-ELECTROPORATION.” The experiments were conducted to separate the two core field-dependent processes according to the main hypothesis. These two processes are defined by the following two critical fields, respectively: (i) the threshold field, H_th_, for MENs to penetrate through the cancer cell membrane to deliver the drug into the cell cytosol (by means of the field-induced localized nano-electroporation effect in the vicinity of MENs); and (ii) the release field, H_r_, that triggers unloading of the drug after the drug-loaded MENs penetrated into the cell. The specificity to the cancer cells was defined not just by the typical HER-2 antibody chemistry but also by the new physical mechanism that relied on the significant difference in the threshold electric field between the healthy and cancer cells. This threshold field was measured to be of the order of 30 Oe and above 200 Oe for the SKOV-3 and HOMEC cells, respectively. Moreover, these experiments indicated that this remote-magnetic-field-triggered electric-field-defined specificity to the cancer cells resulted in a more pronounced eradication of the cancerous cells. The percentage of the cell-penetrated drug was increased by at least a factor of five compared to the traditional antibody-mediated targeting (figure 4). In addition, after the drug was efficiently transferred through the tumor cell membrane by the field-controlled MEN-initiated nano-electroporation, eradication of the majority of the cancer cells (without affecting the healthy cells) was observed within a 24-hour period of a low-energy 30-Oe treatment (figures 5 and 6).

**Figure 1:**
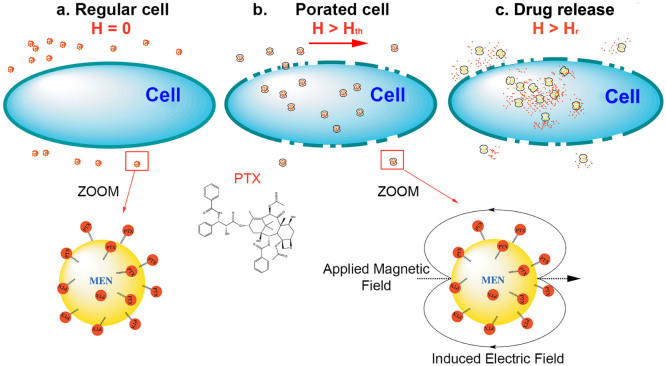
Hypothesis illustration: MENs as field-controlled nano-electroporation sites to let the drug through the cancer cell membranes.

**Figure 2:**
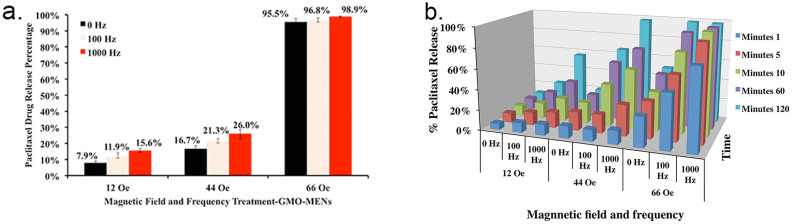
Photo-absorption measurements of the release kinetics.

**Figure 3:**
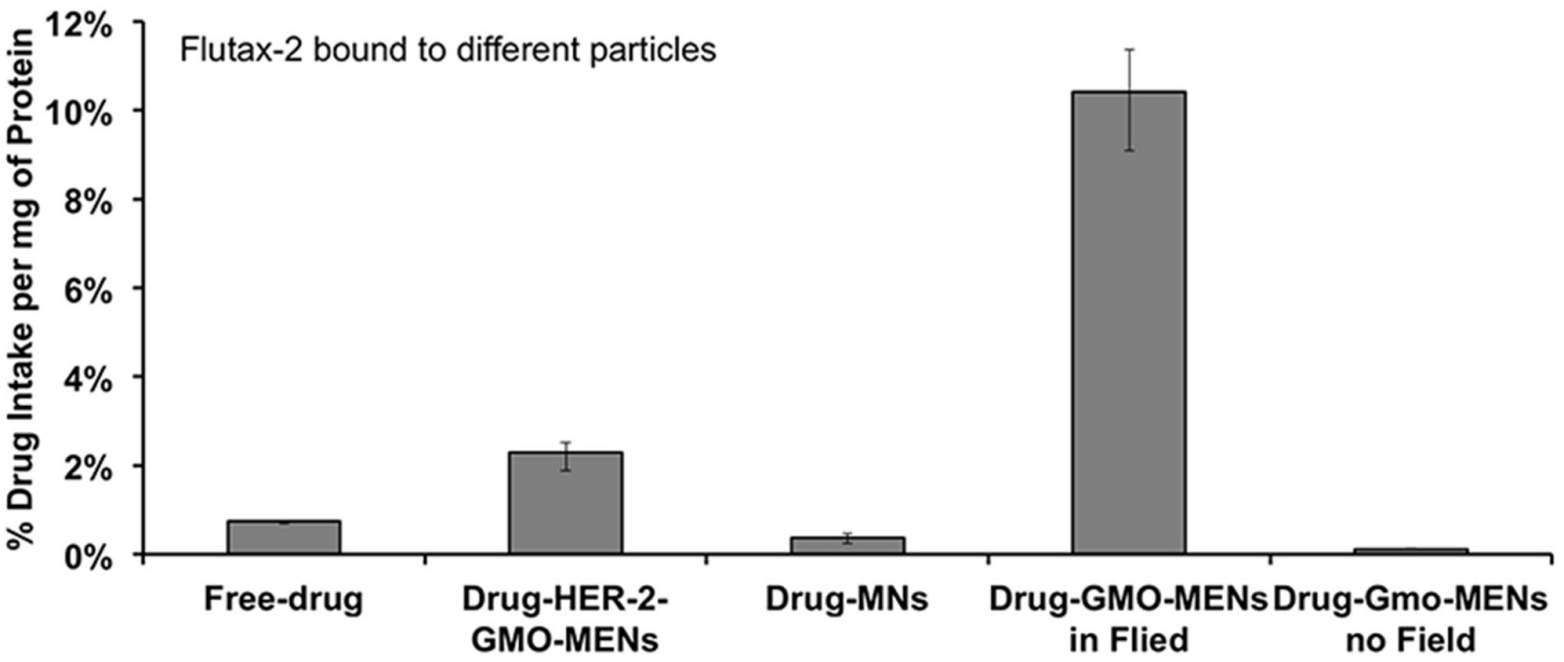
Drug uptake by cancer cells via different carriers.

**Figure 4:**
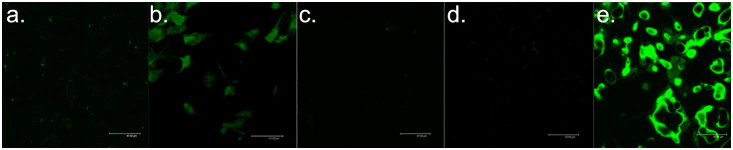
Confocal microscopy imaging of the drug uptake by SKOV-3 with different drug carriers.

To achieve adequately high efficacy of the drug delivery, the value of the release field, H_r_, was chosen to be higher than the value of the threshold field to penetrate through the membrane, H_th_, for the cell of the same type. It can be reminded that the release field is defined by the binding force between the MEN and the drug, while the penetration threshold field, H_th_, is mostly determined by the electric properties of the cell membrane that lead to the localized electroporation effects. We could control the release field by the proper selection of the intermediate layer between the drug and the MEN. (As summarized in Fig. S1, by choosing different intermediate layers we could control the initial release field in a wide range, from less than 10 Oe to over 200 Oe. By default, a 2-nm thin GMO layer was used as the intermediate layer.) In addition, the release field depended on the field treatment duration and the frequency of the a.c. field, as shown in Fig. 2 (bottom). For example, as shown in Fig. 2A, the spectrophotometry measurements of the absorbance at 230 nm (for PTX) indicated that only 1 minute of field treatment at a 66-Oe d.c. magnetic field was sufficient to release over 95% of the drug. As shown in Fig. 2B, the same release efficacy (of over 95%) could be achieved also at an 1000-Hz a.c. field at a smaller field strength of 44 Oe in 2 hours of treatment. This complex dependence can be explained by the fact that the external field effectively reduces the energy barrier that holds together the MEN and the drug while an increase of the treatment duration increases the temperature-induced probability to overcome the barrier or, in other words, break the bond. As for the frequency dependence, in our previous paper we explained the underlying physics through field torque effects that break the bond as the frequency increases^14^. Here, it can be mentioned that although using a.c. external magnetic fields could indeed add another knob to control the targeted delivery, in this study to focus on the proof of the main hypothesis, as illustrated in Fig. 1, we followed the d.c. field scenario. The d.c.-field-controlled drug release kinetics was confirmed also through AFM, FTIR, and Mass Spectrometry, and X-ray diffraction pattern studies, as shown in Fig. S2, Fig. S3, Fig. S4, and Fig. S5, respectively. As confirmed by infrared measurements of the cellular surface temperature (Fig. S8), the MENs's field action didn't trigger any significant temperature changes in the field and frequency range under study. This is in agreement with the fact that the MENs-induced delivery is a relatively energy-efficient process (because of the strong intrinsic magneto-electric coupling) which causes only negligible heat dissipation.

**Figure 5:**
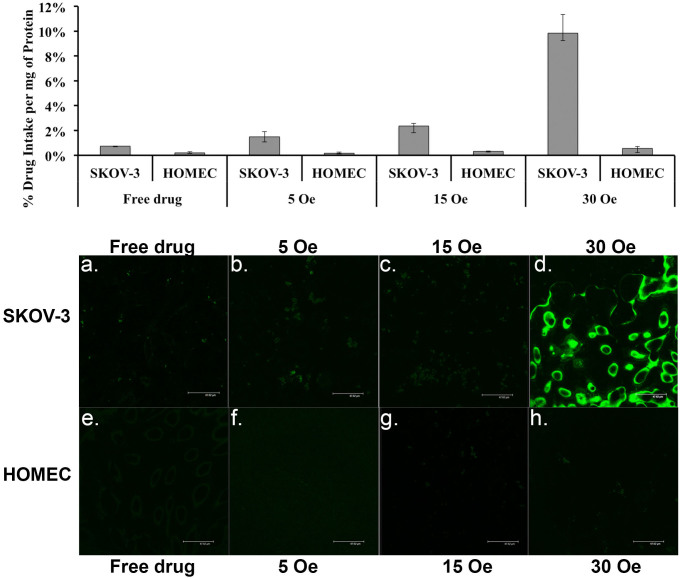
Field dependence of the drug uptake by SKOV-3 and HOMEC cells.

**Figure 6:**
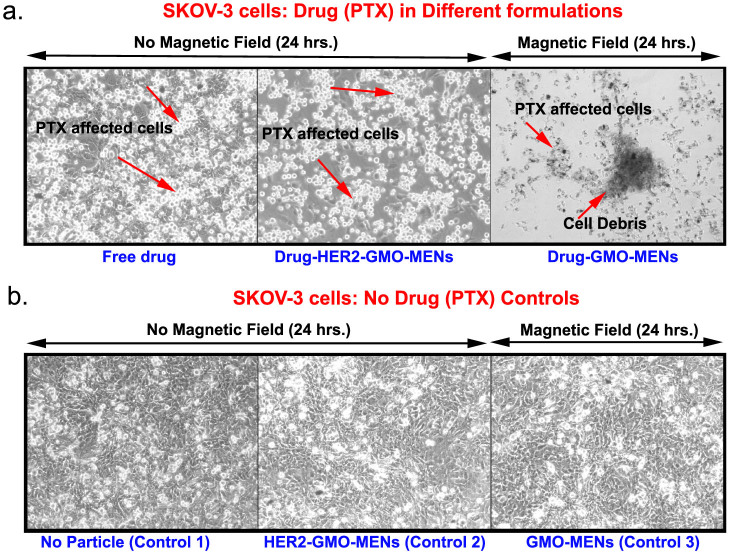
Confocal imaging of SKOV-3 cell viability after treatment by different drug-carrier combinations with and without field.

As for the penetration threshold field, H_th_, we found that for the cancer cell membrane the value was of the order of 30 Oe, i.e. less than the d.c. release field (for the default MEN carriers coated with GMO) of 60 Oe. Again, according to the main hypothesis, to ensure the specificity to the cancer cells, it is important to maintain the remote field above the release value for the tumor cells but lower than the threshold value for the healthy cells. Indeed, at a 30-Oe external d.c. field, the drug couldn't penetrate through the healthy cell membrane for the 24-hour treatment duration, which confirmed that the threshold field for the drug-loaded MENs to penetrate into the healthy cell exceeded 30 Oe during the entire treatment (figure 5). Specifically, the GMO-MENs field-treated HOMEC cells showed negligible drug intake per 1 mg of the cellular protein content. The value was 0.18 ± 0.07, 0.30 ± 0.04, and 0.55 ± 0.16% for the field strength of 5, 15, and 30 Oe, respectively. On the contrary, SKOV-3 cells showed significantly higher values of the drug intake, which was 1.50 ± 0.41, 2.36 ± 0.48, and 10.41 ± 1.54% for the field strength of 5, 15, and 30 Oe, respectively. It can be noted that after a 24-hour 30-Oe field treatment by GMO-MENs, approximately 95 and 34% of HOMEC and SKOV-3 cells, respectively, remained viable (Fig. S6). When the treatment was extended to 36 hours, the percentage of viable cells fell to approximately 85 and 10% for HOMEC and SKOV-3 cells, respectively. These results indicate that further field and frequency optimization could be used to perfect the treatment results.

Least but not last, the cytotoxicity measurements with the standard XTT assay performed on SKOV-3 cells at different concentrations of MENs showed no significant toxicity even at the highest nanoparticles concentration value of 100 μg/ml (figure S6). The chart shows the results of XTT Assay performed on SKOV-3 cells at different concentrations of GMO-MENs.

Finally, the parallel study on MDR cell MES-SA/DX5 proved the applicability of the new nanotechnology to other cancers (figure S8). It might be worth noting that due to the overexpressed transmembrane poteints, e.g. P-glycoprotein, this cell type is known to be relatively impenetrable for many popular chemotherapy drugs, which makes the finding even more significant^31^,^32^.

## Methods

### Preparation of CoFe_2_O_4_-BaTiO_3_ coreshell MENs

CoFe_2_O_4_-BaTiO_3_ core shell MENs were prepared according to the following conventional procedure^33^. As the first step, CoFe_2_O_4_ particles were prepared by the standard hydrothermal method, according to which 0.058 g of Co(NO_3_)_2_.6H_2_0 and 0.16 g of Fe(NO_3_)_3_.9H_2_0 were dissolved in 15 ml of distill water and 0.2 g of polyvinylpyrrolidone was dissolved in 5 ml of aqueous solution containing 0.9 g of sodium borohydride at 120°C for 12 hours. Then, precursor solution of BaTiO_3_ was prepared by mixing 30 ml of aqueous solution containing 0.029 g of BaCO_3_ and 0.1 g of citric acid with 30 ml of ethanolic solution containing 1 g of citric acid and 0.048 ml of titanium (IV) isopropoxide. Coreshell CoFe_2_O_4_-BaTiO_3_ MENs were prepared by mixing 0.1 g of CoFe_2_O_4_ nanoparticles in the BaTiO3 precursor solution and the mixture was sonicated for 2 hrs. Once the CoFe_2_O_4_ nanoparticles were thoroughly dispersed, the mixture was dried on the hot plate at 60°C overnight while continuously stirring. The dried powder was subjected to 780°C for 5 hrs. in a furnace (CMF-1100) and cooled at 52°C per minute to obtained the coreshell MENs of ~30 nm diameter. The particles size distribution was measured using dynamic light scattering method (Malvern-Zetasizer).

### Preparation of GMO-MENs

In-order to load the PTX drug onto the MENs' surface, the nanoparticles were first coated with GMO to adjust the release field at about 30 Oe as required for this application. To achieve this, 1 mg of GMO was added to 5 mg of MENs in 5 ml of the PBS buffer. The mixture was then incubated for 12 hours while being slowly rotated in order to achieve uniform coating. Upon completion of the incubation process, the nanoparticles were centrifuged at 20,000 rpm for 20 minutes at 10°C. The pellet was washed in ethyl acetate:acetone (70:30) solution and re-centrifuged. The washing process was repeated thrice to completely remove the excess unbound GMO. Finally, the obtained pellet was lyophilized for 48 hours and stored for further use. The results of the energy dispersion spectrometry (EDS) that depict the materials composition of GMO-MENs are summarized in Fig. S10.

### Preparation of HER-2 biomarker antibody conjugated GMO-MENs

HER-2 biomarker antibodies were covalently attached onto the GMO-MENs' surface according to the protocol described by Kocbek et al^34^. In-order to covalently attach the HER-2 antibodies, the nanoparticle surface was preliminarily functionalized. For this, 1 mg of GMO-MENs were added to 500 μl of the PBS buffer (pH 7.4). To this solution, 25 μl of N-(3-Dimethylaminopropyl)-N′-ethyl-carbodiimide hydrochloride (EDC) and 25 μl of N-hydroxysuccinimide (NHS), at 1 mg/ml concentration in the PBS buffer (pH 7.4) were added. The solution was incubated for 4 hours at room temperature while being stirred slowly. Then, the sample was centrifuged at 14,000 rpm for 10 minutes at 10°C and the pellet was washed three times with 1 ml of the PBS buffer (pH 7.4). To bind HER-2 antibodies to the functionalized MENs, 10 μl of the antibodies (1 mg/ml) was added to the pellet along with 300 μl of the PBS buffer (pH 7.4). The solution was incubated for 2 hours while being rotated slowly and kept further at 4°C overnight. The solution was centrifuged at 14,000 rpm for 10 minutes at 10°C. The pellet was washed thrice with 1 ml of the PBS buffer (pH 7.4) to remove any excess antibody. The supernatant was collected to determine the amount of the unconjugated HER-2 protein by comparing to the standard plot. A standard calibration plot for HER-2 was obtained by varying the concentration in the range of 1.25–10 μg/ml using Bio-Rad protein assay kit (Braford method) through measuring the absorbance at 595 nm using spectrophotometer Cary-100 (figure S11). The percentage of the conjugated HER-2 was obtained using the following expression: the percentage of HER-2 conjugated = (the total amount of HER-2 added – the amount of the unconjugated HER2 present in the supernatant) × 100. The results indicated that over 70% of the HER-2 antibodies were conjugated to the GMO-MENs' surface.

### Preparation of PTX-MENs, PTX-GMO-MENs and PTX-HER-2-GMO-MENs

After 50 μg of PTX drug was added to the solution of 900 μl of the modified PBS (MPBS) buffer and 100 μl of the desired MEN combination (i.e., MENs, GMO-MENs, and HER-2-GMO-MENs at a 5 mg/ml concentration), the solution was incubated for 3 hours while stirred slowly to obtain uniform binding. Then, the solution was centrifuged at 14,000 rpm for 10 minutes at 10°C to remove any unbounded drug. The supernatant was isolated and absorbance was measured spectrophometrically at 230 nm using Cary-100 UV-VIS spectrophotometer^30^. The PTX loading percentage is shown in Fig. S12. A standard calibration plot for PTX was obtained by varying the drug concentration from 5 to 80 μg in 1 ml of the MPBS solution and the absorbance was measured at 230 nm. The absorption maxima and the standard linear calibration plot of PTX at different drug concentration values are shown in Fig. S13.

### Cell culture experiments

Cell culture experiments were performed using human ovarian carcinoma cell line (SKOV-3) purchased from American Type Culture Collection (Manassas, VA) and were cultured in McCoy's 5A medium (Life Technologies, NY) supplemented with 10% fetal bovine serum (Sigma-Alrich) and 1% penicillin-streptomycin (science-cell). Human Ovarian Microvasicular endothelial cells (HOMEC) from ScienceCell (Carisbad, CA) and were cultured in endothelial cell medium with endothelial cell growth supplement (1%), fetal bovine serum (5%), and penicillin-streptomycin (1%). All the cells were cultured at 37°C cell incubator with a 5% CO_2 _and humidified atmosphere.

### Fluorescence measurements and confocal imaging of drug uptake by SKOV-3 cells for different drug-carrier combinations

Cellular drug uptake measurements and fluorescence imaging were performed using an Oregon Green^®^ 488 paclitaxel (also called Flutax-2). The experiments were performed in dark. For the fluorescence measurements, the SKOV-3 cells were cultured in 24-well plate at a density of 2 × 10^5^ cell per well. After 24-hour incubation at 37°C, the cell medium was replaced with 1 ml/well of the medium containing either one of the four desired drug forms. The concentration of Flutax-2 was normalized to 0.75 μM (1.76 μg/ml) for all the combinations. The cell culture plate was returned to the incubator and incubated for 10 hours. In addition, a set of controls containing no drug for all the combinations was cultured under similar conditions. The cell culture plates containing the Flutax-2-MNs and Flutax-2-GMO-MENs were kept under a 30-Oe field. Upon completion of the 10-hour incubation process, the cells were removed from incubator and the cell culture medium was discarded. The cells were washed with ice-cold PBS buffer thrice. Then, 1 ml of dimethyl sufloxide (DMSO) was added to each well and incubated for 2 hours at 37°C. After two hours, a rubber policeman was used to ensure the complete removal of the attached cells. The solution was centrifuged at 14,000 rpm for 10 minutes at 4°C to obtain the cell lysate. The cell lysate along with the in-taken Flutax-2-GMO-MENs was collected and measured for the fluorescence of Flutax-2 (using BioTek instruments, synergy HT) at λ_ex_ = 496 nm and λ_em_ = 524 nm to determine the concentration. All the fluorescence measurements were recorded by subtracting the corresponding controls to adjust the background fluorescence from the cellular components. The protein content of the cell lysate was determined using Bio-Rad protein assay kit (Braford method) by measuring the absorbance at 595 nm using a Cary 100 UV-VIS spectrophotometer.

As for the imaging studies, the cells were cultured on glass cover slips (1 × 1 in^2^) pre-coated with the poly-L-Lysine (used as a cell adhesion promoter) in a 6-well cell culture plate at a density of 5 × 10^4^ were cultured and let rested for about 10 minutes. Then, 2 ml of Cell medium was added along the walls of the wells. The cell culture plate was incubated for 24 hours at 37°C. The cells were supplied with the fresh cell medium that contained either of the four drug forms under study normalized to the 0.75 μM (1.76 μg/ml) of Flutax-2 concentration. The cell plate containing the Flutax-2-GMO-MENs was kept under a 30-Oe magnetic field. The cell plates were incubated for 10 hours at 37°C. After the incubation process, cover slips were washed three times with the PBS buffer and fixed with 4% paraformaldehyde for 30 minutes followed by washing thrice with the PBS buffer. The cover slips were mounted onto a glass slide using a mounting medium (ProLong Gold Antifade Reagent). The excess mounting medium was removed by placing a small piece of Whatman paper around the edges. After the samples were dried for 2 hours, they were imaged through confocal microscopy (TCS SP2, Leica Microsystems, Germany) at 488 nm (100%) illusion of an argon-ion laser using 60× oil immersion objectives with a high numeric aperture and 1× confocal electronic zoom settings to visualize cells. Flutax-2 calibration plot is shown in Fig. S14.

## Author Contributions

S.K. designed, oversaw, and supervised the entire project. R.G. conducted all the key measurements including AFM, FTIR, spectrophotometry, cell preparation, in-vitro studies of the targeted delivery/release, field-controlled uptake by specific cells, pharmacokinetics and cytotoxicity measurements. P.L. helped design the magnetic control systems. C.R. advised on the ovarian cancer studies. M.N. advised on the cancer immunology aspects of the research. V.A. helped with the fluorescence studies.

## Supplementary Material

Supplementary Information Supplementary Information
