# Supplementary material for: Magneto-electric Nanoparticles to Enable Field-controlled High-Specificity Drug Delivery to Eradicate Ovarian Cancer Cells
Source: Sci Rep. 2013 Oct 16;3:2953. doi: 10.1038/srep02953 (PMC3797424; doi:10.1038/srep02953)
Supplement: Supplementary Information [file srep02953-s1.pdf]

SUPPLEMENTARY INFORMATION FILE

Title: Magneto-electric Nanoparticles to Enable Field-controlled High-Specificity Drug Delivery to Eradicate Ovarian Cancer Cells

Authors: Rakesh Guduru[1,2], Ping Liang[3], Carolyn Runowicz[1,4], Madhavan Nair[1], Venkata Atluri[1], and Sakhrat Khizroev[1,2,3*]

Affiliations
[1]Center for Personalized NanoMedicine, Herbert Wertheim College of Medicine, Florida International University, Miami, Florida 33199
[2]Electrical and Computer Engineering, College of Engineering, Florida International University, Miami, Florida 33174
[3]Electrical Engineering, University of California, Riverside, CA 92521
[4]Department of Obstetrics and Gynecology, Herbert Wertheim College of Medicine, Florida International University, Miami, Florida 33199

*To whom the correspondence should be addressed:
Sakhrat Khizroev, Director, Center for Personalized Nanomedicine
Department of Immunology, Herbert Wertheim College of Medicine, Florida International University, Miami, FL, Ph. 305-348-3724, E-mail: khizroev@fiu.edu

SUPPLEMENTARY INFORMATION FIGURE LEGENDS

Figure S1: The dependence of the release field on the intermediate layer material.
Figure S2: Atomic force microscopy.
Figure S3: Fourier transform infra-red studies.
Figure S4: Mass spectrometry.
Figure S5: XRD analysis.
Figure S6: Confocal microscopy for 24- and 36-hour 30-Oe-field treatments
Figure S7: XTT cytotoxicity assay.
Figure S8: Surface temperature.
Figure S9: Drug uptake by MDR cells.
Figure S10: Energy dispersion spectroscopy of MENs.
Figure S11: Standard HER-2 antibody concentration calibration curve.
Figure S12: PTX drug loading percentages.
Figure S13: PTX calibration plot.
Figure S14: Flutax-2 calibration plot.

SUPPLEMENTARY INFORMATION TABLE LEGENDS

Table S1. The field release dependence on the field, frequency, and the treatment duration.
Table S2. Trypan-Blue cell viability assay.
Table S3. Size and Zeta-potential measurements.

Supplementary Information Figures

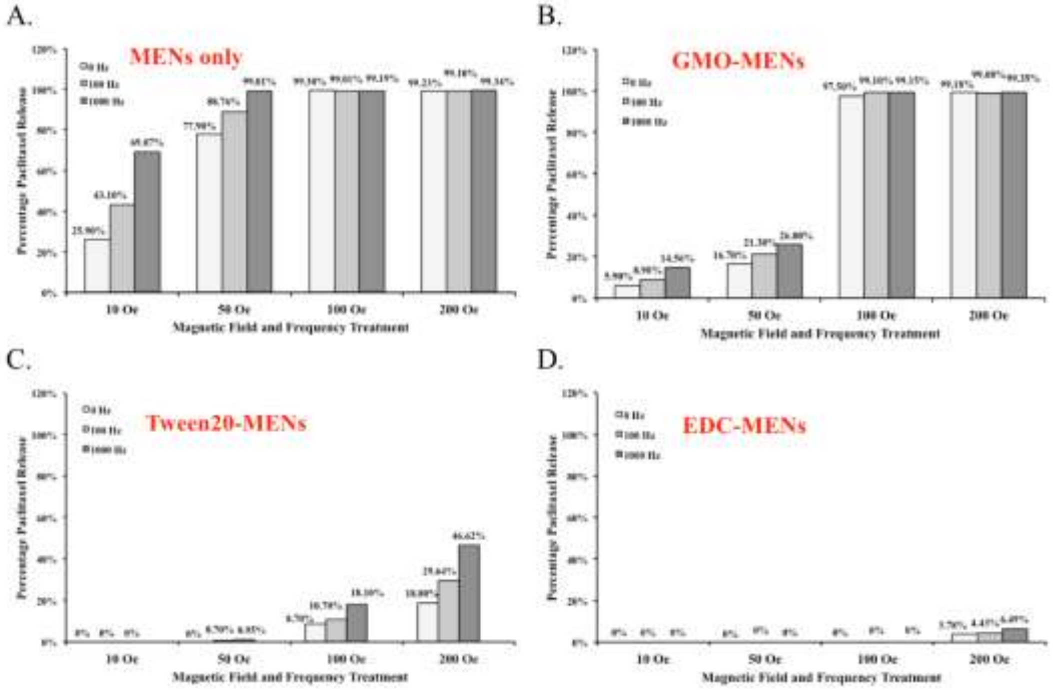

Figure S1: **The dependence of the release field on the intermediate layer material.** The four charts show the release kinetics (field strength and frequency dependence on the field treatment duration) for (A) uncoated MENs, (B) GMO-MENs, (C) Tween20-MENs, and (D) EDC-MENs. Here EDC stands for 1-Ethyl-3-(3-dimethylaminopropyl)carbodiimide

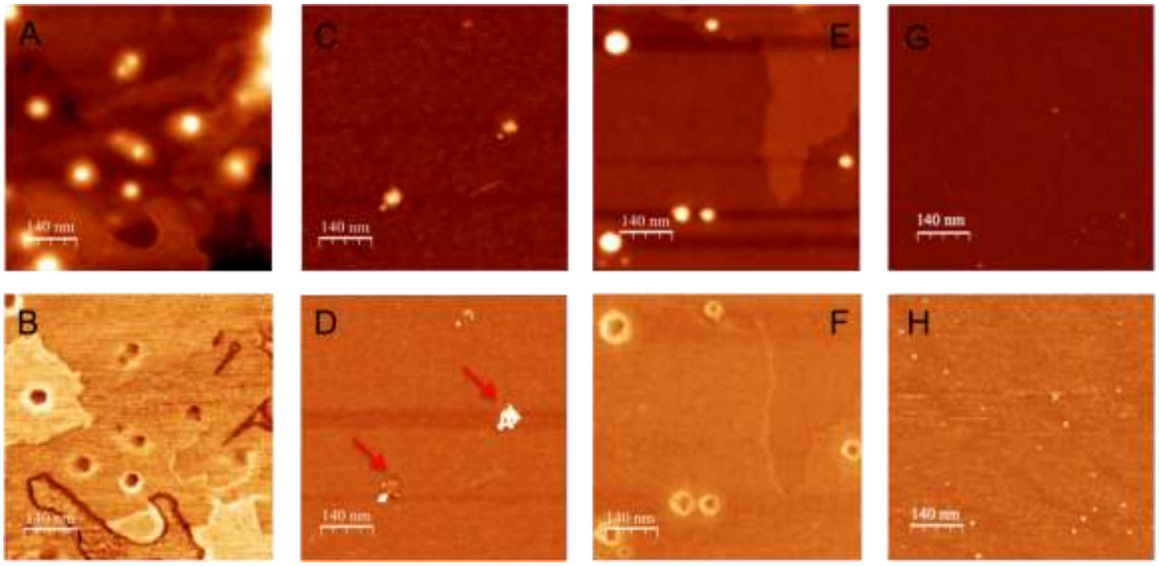

Figure S2: Atomic force microscopy. Topography and phase-contrast atomic force microscopy (AFM) images of (**A and B**) GMO-MENs only, (C and D) GMO-MENs loaded with PTX, (**E and F**) GMO-MENs after the 36-Oe 1000-Hz field-triggered release, and (**G and H**) PTX only.

The imaging was performed in Tapping Mode™ using Nanoscope IIIa Multimode AFM (Veeco Metrology, CA). The samples were specially prepared for the imaging by dispersing desired MENs on the silicon wafer surface (1 × 1 cm^2). To achieve uniform dispersion of MENs, the nanoparticles of choice were first mixed in chloroform solution (1mg of particles in 600 µl of chloroform). Then, 100 µl of this solution was mixed with 200 µl of isopropanol and the solution was set to 3500-rpm centrifugation for 3 minutes. Finally, the obtained pellet of MENs was dissolved in 200 µl of Chloroform and 20 µl of this solution was placed on a silicon wafer and then dried at room temperature overnight. The silicon wafer containing the desired MENs were glued on to a metallic sample puck and mounted on the AFM stage. All the images were performed in 256 × 256 scan resolution at a scan rate of 0.5 Hz at a 40-nm Z-scale and a 60^0-Phase angle.

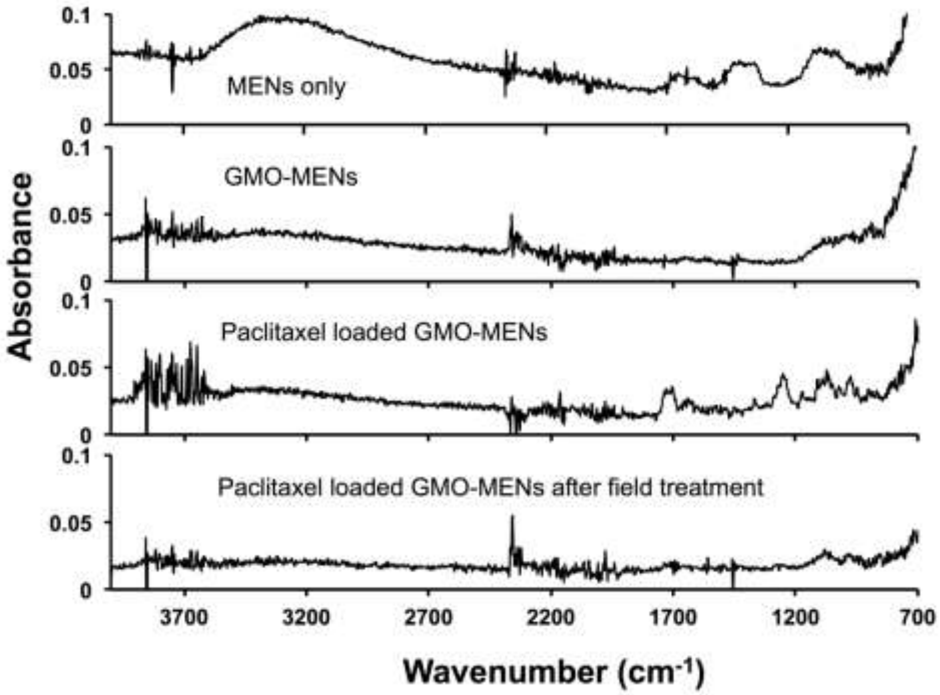

Figure S3: Four transform infra-red studies. Fourier Transform Infra-Red **(**FTIR) results showing the spectrum change at different stages of the drug release.

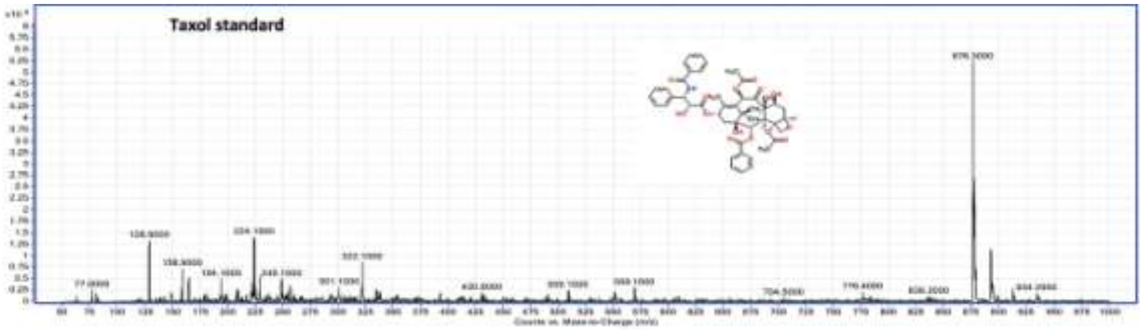
Taxol standard

A.

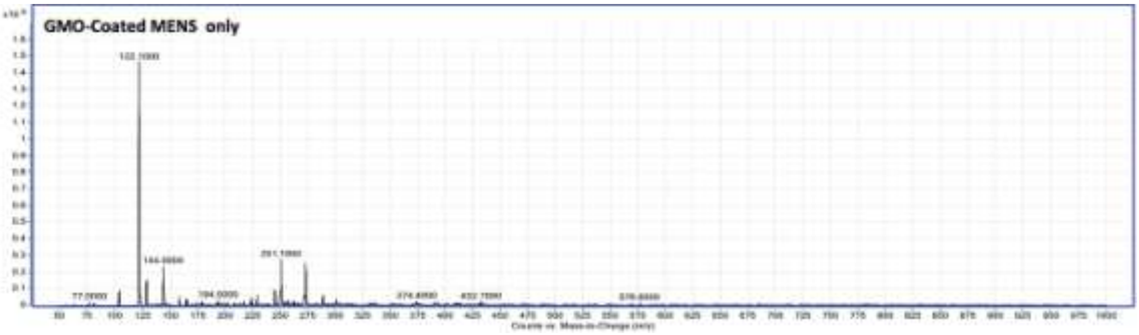
GMO-Coated MENS only

B.

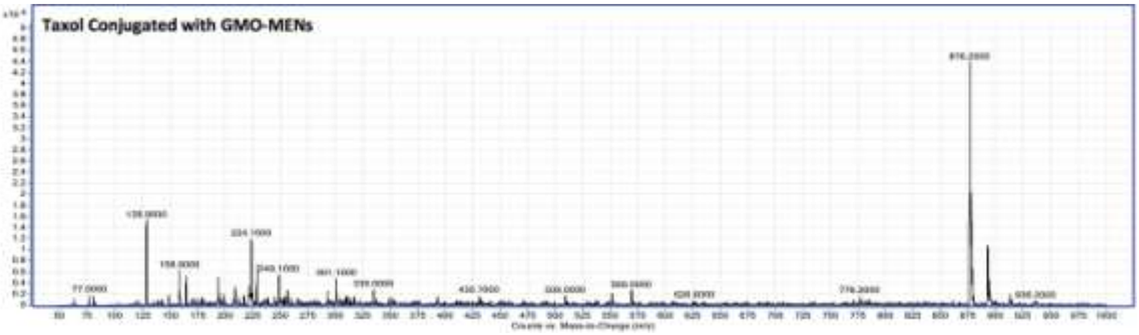
Taxol Conjugated with GMO-MENs

C.

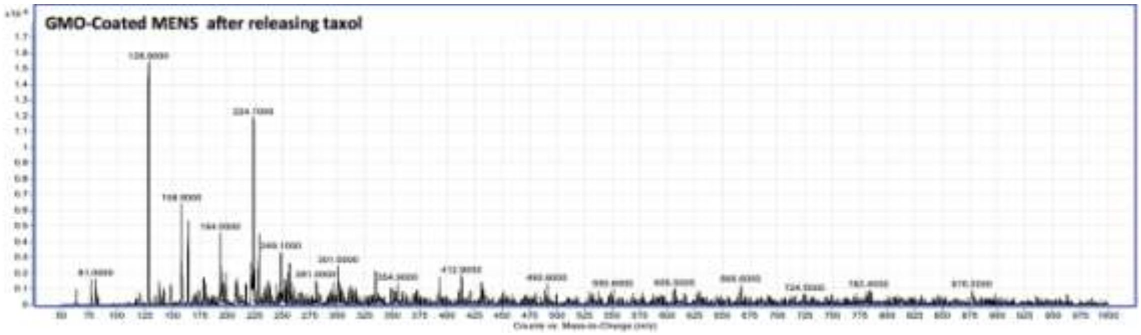
GMO-Coated MENS after releasing taxol

D.

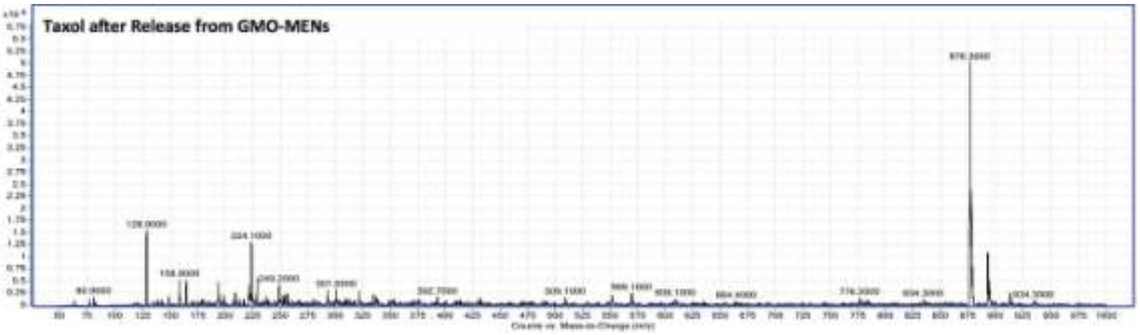
Taxol after Release from GMO-MENs

E.

Figure S4: **Mass spectrometry**. Mass spectroscopy results showing (A) the significant peak of PTX at 876 nm in the MBPS buffer, (B) GMO-MENs in the MPBS buffer, (C) the significant peak of PTX at 876 nm when conjugated to the GMO-MENs in the MPBS buffer, (D) PTX-GMO-MENs after the field treatment, (E) PTX after the release.

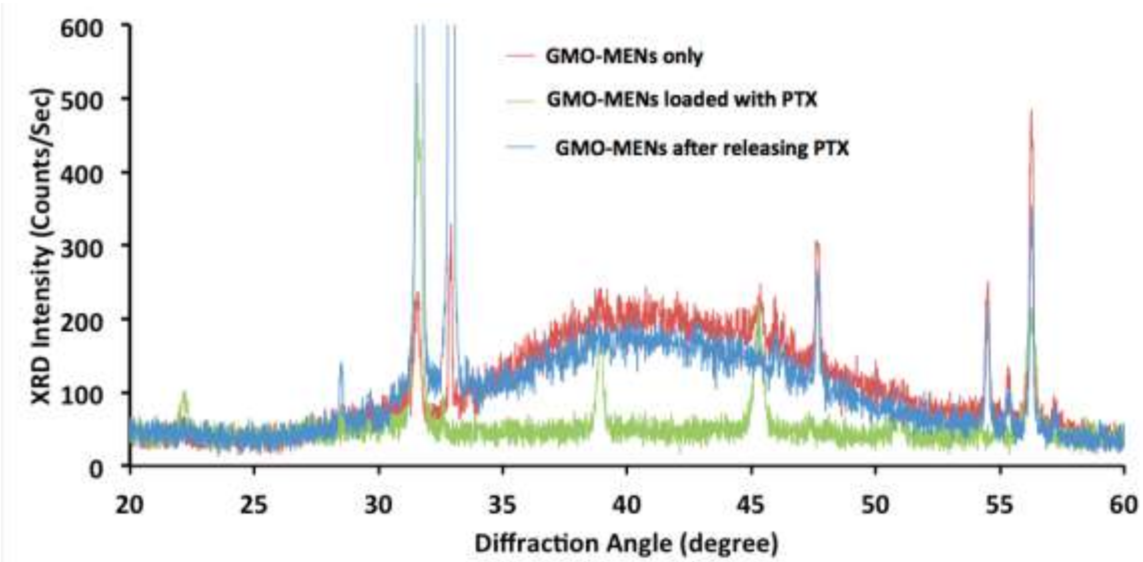

Figure S5: **XRD pattern**. XRD results of GMO-MENs at the three important phases of the release: (RED) before and (GREEN) after binding with PTX before the release and (BLUE) after the field-triggered release. Prior to performing the measurements, the nanoparticles were dispersed on a Silicon wafer and dried for 24 hours.

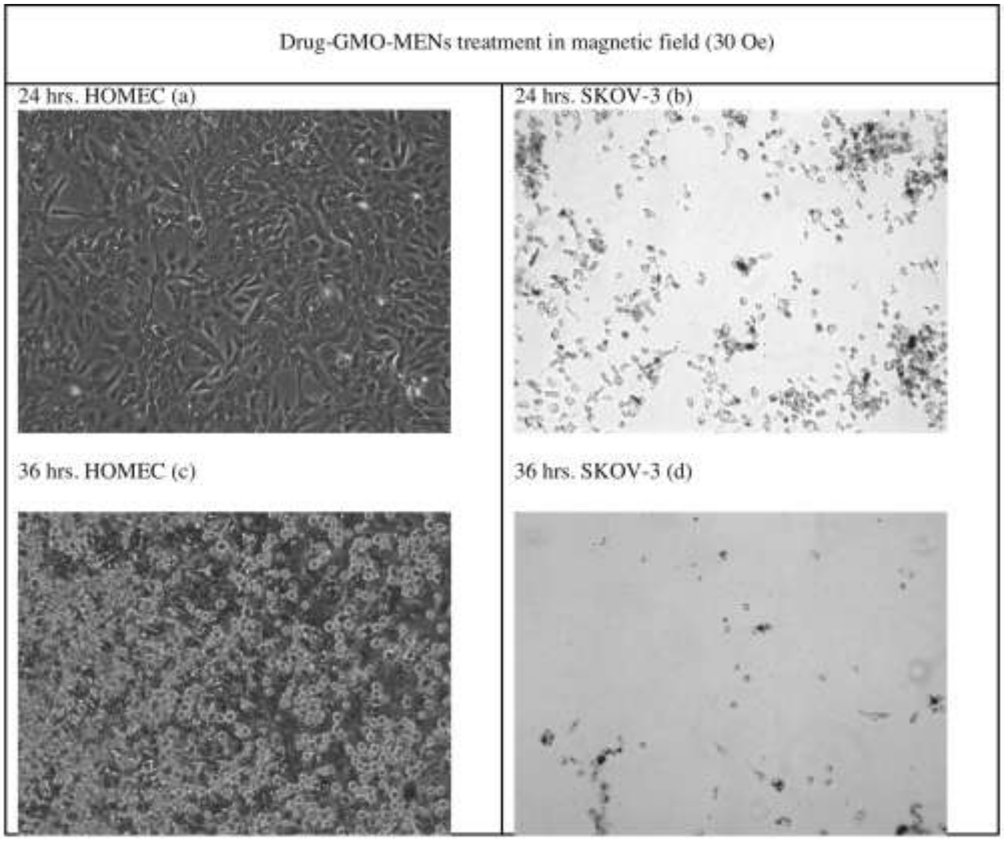

Figure S6: **Confocal microscopy for 24- and 36-hour 30-Oe-field treatment**. Confocal images of: (a) HOMEC Cells treated with PTX drug loaded onto GMO-MENs in a 30-Oe field for 24 hrs. (b) SKOV-3 Cells treated with PTX drug loaded onto GMO-MENs in a 30-Oe field for 24 hrs. (c) HOMEC Cells treated with PTX drug loaded onto GMO-MENs in a 30-Oe field for 36 hrs. (d) SKOV-3 Cells treated with PTX drug loaded onto GMO-MENs in a 30-Oe field for 36 hrs.

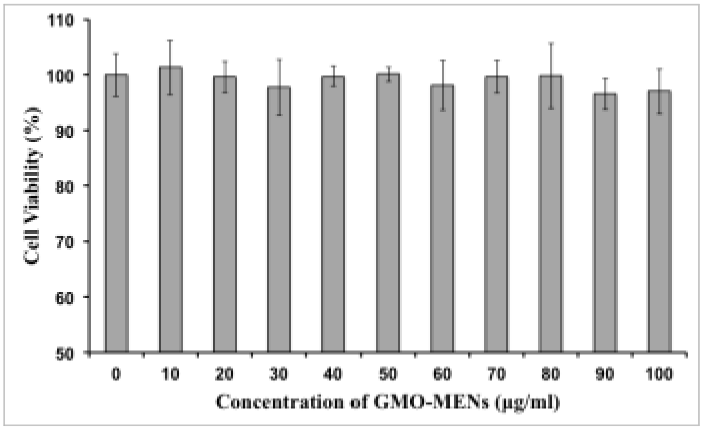

Figure S7: XTT cytotoxicity assay. The chart shows the results of XTT Assay performed on SKOV-3 cells at different concentrations of GMO-MENs (n=3).

A. SKOV-3 before and after H

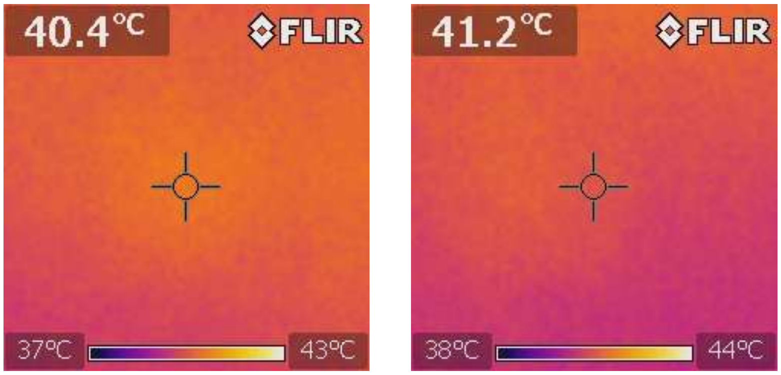

B. HOMEC before and after H

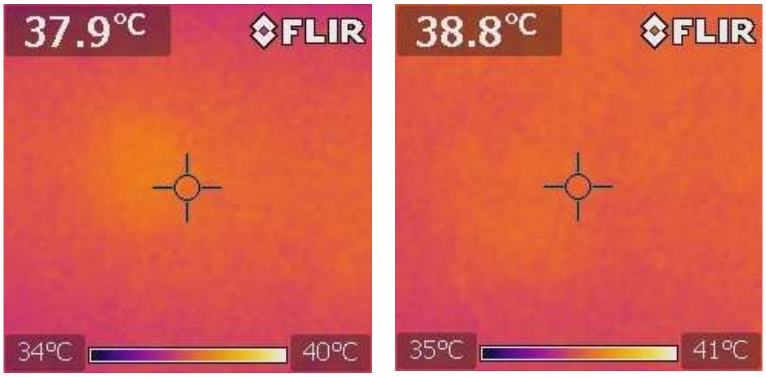

Figure S8: **Surface temperature**. Surface temperature measured via Infra-red (IR) light for (A) cancerous (SKOV-3) and (B) healthy (HOMEC) ovarian cells before and 100-Oe field treatment

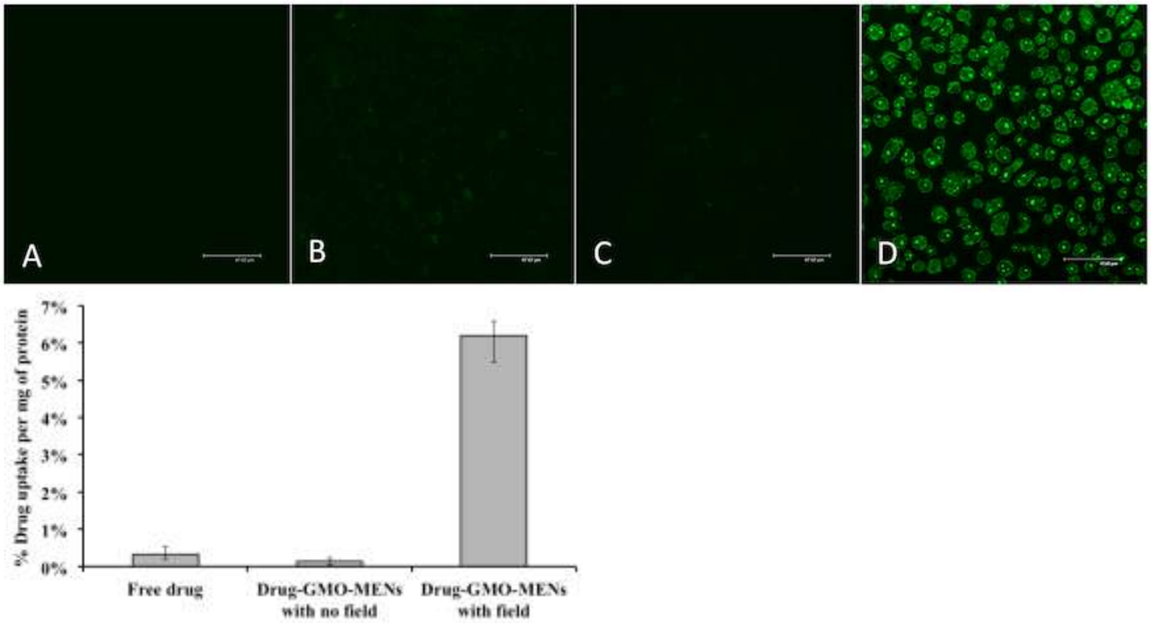

Figure S9: Drug uptake by MDR cells. Confocal microscopy imaging of the uptake of Flutax-2 by MDR cell MES-SA/DX5 for four different drug-delivery-system combinations: (a) no drug, (b) free Flutax-2, (c) Flutax-2-GMO-MENs with no field. (d) Flutax-2-GMO-MENs with 30 Oe field. The bottom insert shows the chart that summarizes the quantitative results.

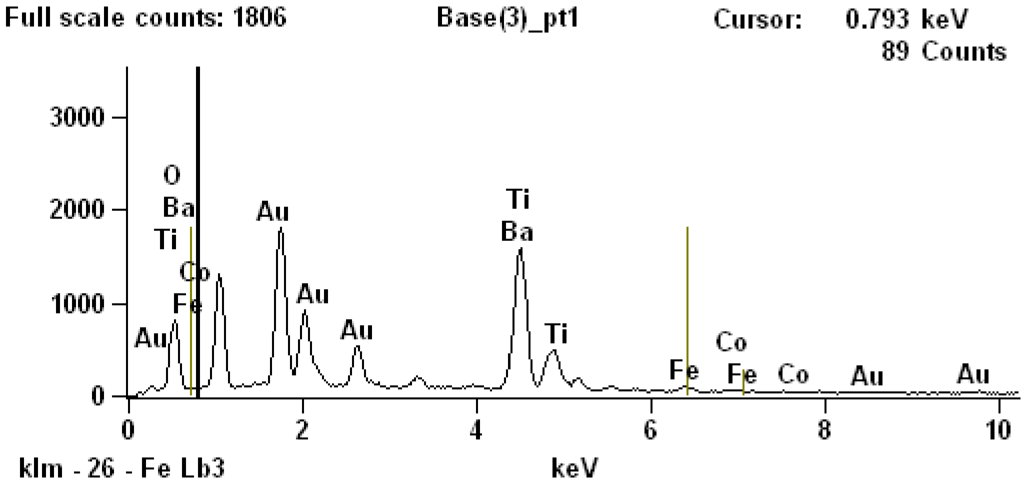

a.

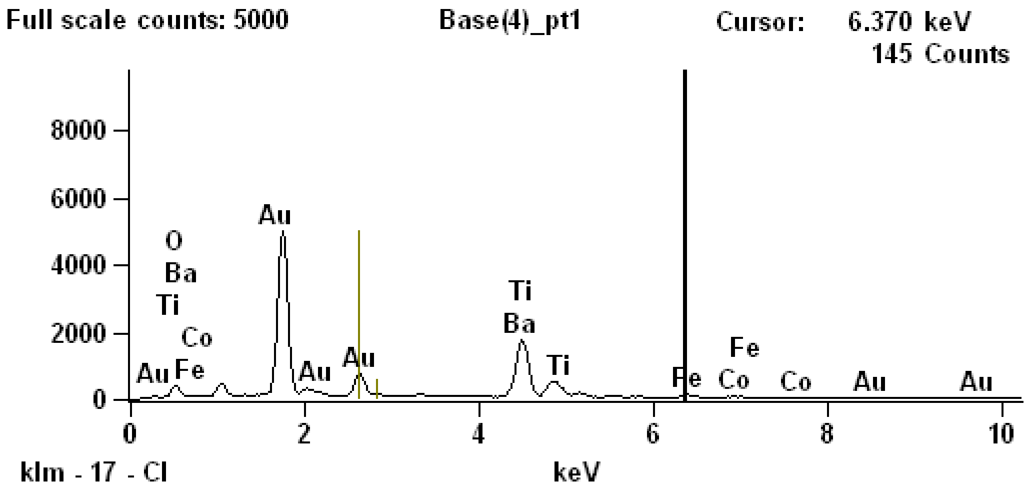

b.

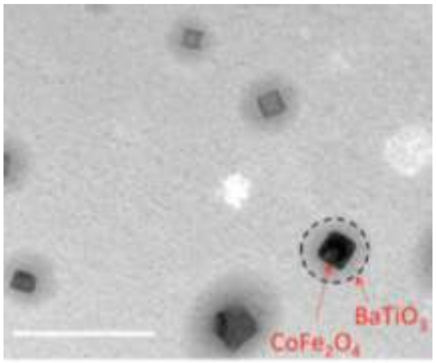

c.

Figure S10: **Energy dispersion spectroscopy of MENs**. Energy dispersion spectroscopy (EDS) results depict the composition of **(a)** MENs only and **(b)** GMO-coated MENs. (c) TEM image showing a two-phase core-shell nature of the 30-nm MENs. The scale bar length is 100 nm.

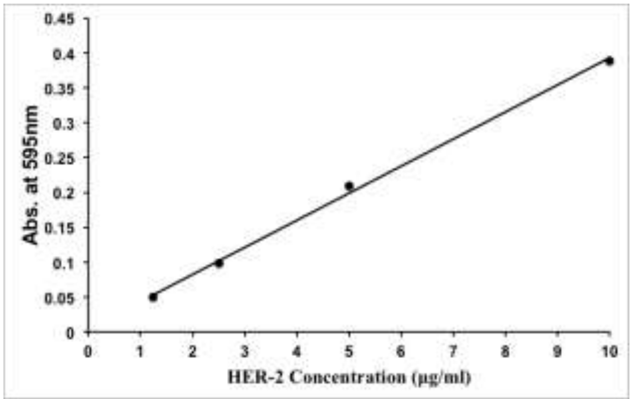

Figure S11: **Standard HER-2 antibody concentration calibration curve**. Standard HER-2 Antibody plot was obtained using Bio-Rad protein assay kit at 595 nm absorbance.

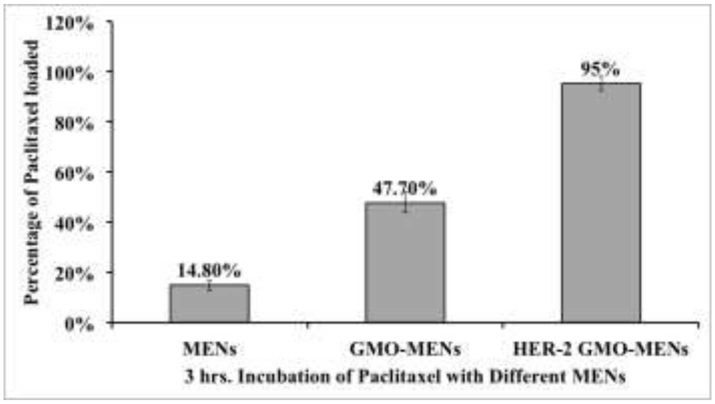

Figure S12: **PTX drug loading percentages.** PTX drug loading percentage for MENs, GMO-MENs and HER-2-GMO-MENs after 3 hrs. incubation (n=3).

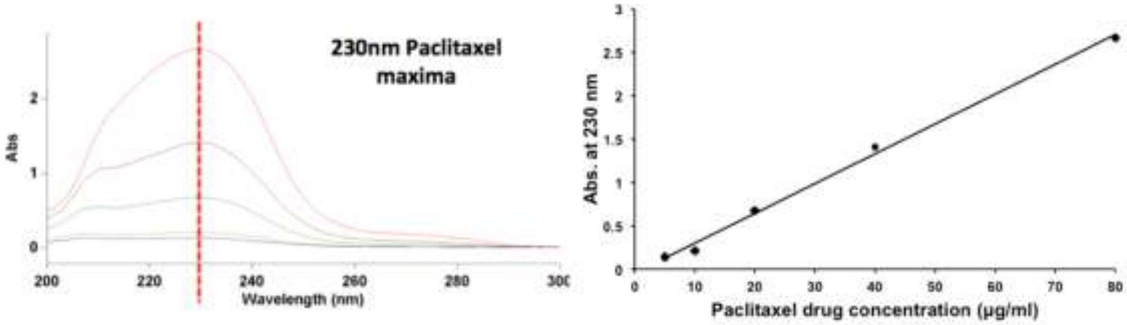

Figure S13: **PTX calibration plot.** The absorption maxima and the standard linear calibration plot for PTX at different drug concentration values determined spectrophotometrically.

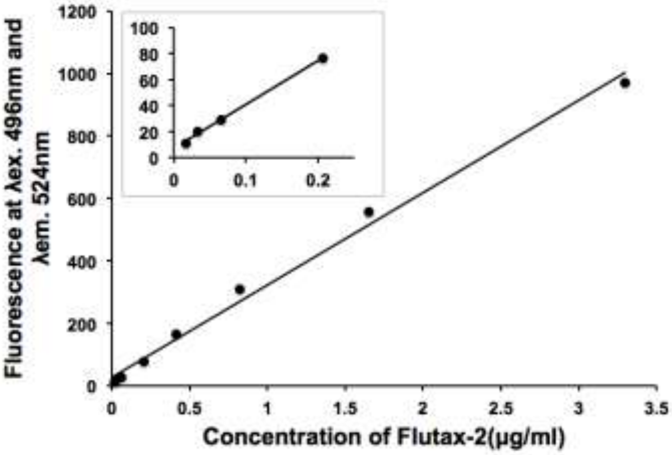

Figure S14: **Flutax-2 calibration plot.** Standard linear calibration plot for Flutax-2 at different concentration was determined using flurometer at excitation wavelength of 496 nm and emission wavelength of 524 nm.

Supplementary Information Tables

Table S1: The field release dependence on the field, frequency, and the treatment duration. The table shows the percentage of the field-triggered released PTX at different combinations of the magnetic field strength (12, 44, and 66 Oe) and frequency (0, 100, and 1000 Hz) and the treatment duration (1, 5, 10, 60, and 120 minutes).

Field Strength	Frequency	Percentage Paclitaxel Release at Different Treatment Time Durations (Minutes)				
		1	5	10	60	120
12 Oe	0 Hz	6.1%	9.1%	10.3%	11.4%	11.1%
	100 Hz	9.5%	12.8%	15.3%	20.5%	24.4%
	1000 Hz	10.7%	15.4%	22.6%	33.7%	56.7%
44 Oe	0 Hz	11.8%	18.3%	20.9%	22.2%	21.1%
	100 Hz	11.2%	18.5%	41.3%	57.9%	66.2%
	1000 Hz	13.4%	30.4%	58.2%	73.2%	97.8%
66 Oe	0 Hz	29.1%	36%	38.4%	49.8%	50.5%
	100 Hz	53.1%	62.9%	78.5%	92.3%	98.5%
	1000 Hz	78.1%	93.7%	98.5%	98.2%	98.2%

Table S2: Trypan-Blue cell viability assay. The table shows the percentage of SKOV-3 cells viable after the 24-hour field-treatment period for different PTX-MEN combinations and field treatment conditions (n=3). For comparison, in 24 hours, approximately 95% of HOMEC cells remain viable after the equivalent treatment by GMO-MENs at a 30-Oe field. When the treatment was extended to 36 hours, the percentage of viable cells fell to approximately 10 and 85% for SKOV-3 and HOMEC cells, respectively.

Drug	Field (30 Oe)	Type of cell	Type of Treatment	Percentage Cells Alive
-	-	SKOV-3	Control-1 (No particle)	99 ± 1 %
	-	SKOV-3	Control-2 (GMO-MENs-HER2)	98 ± 1%
	+	SKOV-3	Control-3 (GMO-MENs)	98.5 ± 0.7 %
+	-	SKOV-3	FREE	86 ± 8.8%
	-	SKOV-3	GMO-MENS-HER2	71 ± 9.8%
	+	SKOV-3	GMO-MENS	31 ± 11.8%
	+	SKOV-3	GMO-MENs	33.8 ± 9.3% (24 hrs.)<br>9.7 ± 4.1% (36 hrs.)
	+	HOMEC	GMO-MENs	94.9 ± 2.4% (24 hrs.)

				84.1 ± 7.8% (36 hrs.)

Table S3. Size and Zeta-potential measurements. The Malvern Zeta-sizer was used to measures size and Zeta-potential of the MENs, GMO-MENs, HER2-GMO-MENs, and PTX-GMO-MENs (n=3). Concentration of the nanoparticles used for the measurements was 500ug/ml of D.I water. Note: The measurements represented are the average of three independent measurements.

Type of measurement	Type of Particles			
	MENs	GMO-MENs	HER2-GMO-MENs	PTX-GMO-MENs
Size (nm)	28.6 ± 7.5	30.9 ± 8.6	43.0 ± 3.6	44 ± 6.6
Zeta-Potential (mV)	-45.0 ± 1.7	-41.6 ± 0.3	-26.3 ± 0.4	-40.7 ± 0.1
